# AIDSVu Cities’ Progress Toward HIV Care Continuum Goals: Cross-Sectional Study

**DOI:** 10.2196/49381

**Published:** 2024-02-26

**Authors:** Nicole Hood, Nanette Benbow, Chandni Jaggi, Shamaya Whitby, Patrick Sean Sullivan, AIDSVu Health Department Collaborators

**Affiliations:** 1 Department of Epidemiology Rollins School of Public Health Emory University Atlanta, GA United States; 2 Department of Psychiatry and Behavioral Sciences Feinberg School of Medicine Northwestern University Chicago, IL United States; 3 See Acknowledgements Atlanta, GA United States

**Keywords:** HIV, epidemiology, surveillance, HIV care continuum, cities, HIV public health, HIV prevention, diagnosis, HIV late diagnosis

## Abstract

**Background:**

Public health surveillance data are critical to understanding the current state of the HIV and AIDS epidemics. Surveillance data provide significant insight into patterns within and progress toward achieving targets for each of the steps in the HIV care continuum. Such targets include those outlined in the National HIV/AIDS Strategy (NHAS) goals. If these data are disseminated, they can be used to prioritize certain steps in the continuum, geographic locations, and groups of people.

**Objective:**

We sought to develop and report indicators of progress toward the NHAS goals for US cities and to characterize progress toward those goals with categorical metrics.

**Methods:**

Health departments used standardized SAS code to calculate care continuum indicators from their HIV surveillance data to ensure comparability across jurisdictions. We report 2018 descriptive statistics for continuum steps (timely diagnosis, linkage to medical care, receipt of medical care, and HIV viral load suppression) for 36 US cities and their progress toward 2020 NHAS goals as of 2018. Indicators are reported categorically as met or surpassed the goal, within 25% of attaining the goal, or further than 25% from achieving the goal.

**Results:**

Cities were closest to meeting NHAS goals for timely diagnosis compared to the goals for linkage to care, receipt of care, and viral load suppression, with all cities (n=36, 100%) within 25% of meeting the goal for timely diagnosis. Only 8% (n=3) of cities were >25% from achieving the goal for receipt of care, but 69% (n=25) of cities were >25% from achieving the goal for viral suppression.

**Conclusions:**

Display of progress with graphical indicators enables communication of progress to stakeholders. AIDSVu analyses of HIV surveillance data facilitate cities’ ability to benchmark their progress against that of other cities with similar characteristics. By identifying peer cities (eg, cities with analogous populations or similar NHAS goal concerns), the public display of indicators can promote dialogue between cities with comparable challenges and opportunities.

## Introduction

Public health surveillance data are foundational in the understanding of the current state of the HIV and AIDS epidemics [1]. Surveillance data provide significant insight into patterns within and progress toward achieving targets for each of the steps in the HIV care continuum—here, defined as timely diagnosis of HIV infection, linkage to HIV medical care, receipt of HIV medical care, and HIV viral load suppression—for geographic areas and populations [2]. Dissemination of these data can be used to prioritize certain steps in the continuum, geographic locations, and groups of people.

AIDSVu was created in 2009 as part of a cooperative private-public-academic partnership between Emory University’s Rollins School of Public Health, Gilead Sciences, Inc, and the Center for AIDS Research at Emory University [3]. AIDSVu aims to provide policy makers, health departments, and others who have a stake in health with an instrument to educate the public, monitor progress and trends, and judiciously advocate for resources and plan programs. Here, we report, for the first time, descriptive statistics for 36 US cities’ status for each of the HIV care continuum steps and their progress toward achieving 2020 National HIV/AIDS Strategy (NHAS) goals based on AIDSVu data [4]. We describe a standardized method to compare HIV care continuum data at the city, regional, and national levels.

## Methods

### Data Collection

We obtained the data used in this analysis through a data request in the spring of 2020 sent to AIDSVu-participating health departments for continuum indicators for people with HIV aged 13 years and older from the enhanced HIV/AIDS reporting system. The request was sent to health departments who had submitted data to AIDSVu in the past or who expressed interest in participating. A total of 36 cities provided stratified data without identifiers related to timely HIV diagnosis, linkage to HIV care, receipt of HIV care, and HIV viral suppression. Data provided on the AIDSVu website are regularly updated and city participation varies by year; these analyses use only the data as they were available for 2018.

In this study, we defined cities as single cities, single or multiple counties that included the named city, or metropolitan statistical areas (MSAs) that included the named city. Use of common SAS code to calculate indicators enabled comparability of indicators across jurisdictions. Data stratifications for display (ie, by age, sex, and race or ethnicity) were chosen by the jurisdictions. For stratification purposes, age was defined as age at initial diagnosis for timely diagnosis and linkage to care; age for the other HIV continuum measures was age as of the end of 2018.

Although AIDSVu HIV care continuum indicator definitions were guided by a process that involved the Centers for Disease Control and Prevention (CDC), the counts and percentages for each of the 4 HIV care continuum indicators were defined slightly differently from those of the CDC (see the AIDSVu website for more information) [3,5]. Timely HIV diagnoses were assessed among persons aged 13 years and older with new HIV diagnoses within the 5-year period from 2014 through 2018. An HIV diagnosis was considered timely if the person did not receive an AIDS diagnosis within 3 months of their initial HIV diagnosis. Timely diagnosis represented the inverse of the traditional late diagnosis continuum measure. Linkage to care was defined as having a CD4 lymphocyte or HIV viral load test within 1 month of diagnosis among those at least 13 years of age with a new HIV diagnosis in the 5-year period from 2014 to 2018. The numerator for the receipt of care definition included individuals who were 13 years of age or older and had been diagnosed with HIV by the end of 2017 and were alive throughout 2018 with ≥1 CD4 lymphocyte or HIV viral load test within 2018. The denominator included those living with HIV in 2018 (excluding those newly diagnosed in 2018). Those diagnosed with HIV by the end of 2017 and living with HIV throughout 2018 with an HIV viral load <200 copies/mL for their most recent HIV viral load test within 2018 were determined to be virally suppressed.

### Data Analysis

Descriptive analyses were conducted for data as of 2018 from 36 cities using SAS (version 9.4, SAS Institute). Cities were categorized by US Census region (ie, Midwest, 2 cities; Northeast, 9 cities; South, 21 cities; and West, 4 cities). The national HIV case counts and percentages for timely diagnosis, receipt of care, and viral suppression were obtained from the CDC’s HIV Surveillance Report for 2018 [6]. We compared indicators reported by the cities with NHAS-designated HIV care continuum benchmarks proposed to be achieved by 2020 [4]. There is currently an updated set of goals through 2025 [7]; however, because our data are from 2018, we compared the data to 2020 goals. Given that there is not an NHAS indicator for timely diagnosis, we used the goal for the indicator called “Knowledge of HIV+ Status,” which was 90% (note: “Knowledge of HIV+ Status” is not tied to a particular time period of receipt of diagnosis) [4]. The NHAS linkage to care goal was to have 85% of persons with a new HIV diagnosis linked to HIV medical care within 1 month of diagnosis. This goal was applied to our linkage to care indicator. The NHAS retention in care goal of 85% was adapted for our receipt of care indicator, and the NHAS viral suppression goal of 80% was used for our viral suppression indicator.

The cities’ progress toward each of the HIV care continuum goals was assessed by determining the relative percentage difference from each goal. For example, the NHAS goal for linkage to care was 85% and the percentage of people in Atlanta with newly diagnosed HIV linked to an HIV care provider was 69% (n=5321). To calculate Atlanta’s progress, we subtracted 0.69 from 0.85, divided the resulting number by 0.69, and then multiplied it by 100 to say that Atlanta was within 25% of achieving the NHAS goal for linkage to care. We categorized city- and target-specific progress toward the goal as follows: met or surpassed the goal, within 25% of attaining the goal, or further than 25% from achieving the goal. We applied the same method to each of the priority populations for the individual cities’ progress tables. We defined priority populations as sex, race or ethnicity, and age groups. Finally, we categorized age as the following: 13-24, 25-44, 45-59, and 60 years of age and older. We calculated the percentage of cities that were at least within 25% of meeting each indicator goal within priority populations.

### Ethical Considerations

As this study used publicly available, secondary, deidentified data (numbers were aggregated to the city level or beyond), ethical evaluation via institutional review board review was not warranted.

## Results

### All Cities’ HIV Care Continuum Indicators

Within our 36 cities, 447,371 people were living with HIV as of 2018, with 82,827 people newly diagnosed with HIV during the 5-year period from 2014 to 2018. In 2018 alone, 15,767 people were newly diagnosed with HIV. Table 1 displays the numerators and percentages for HIV care continuum indicators at the national (except for linkage to care), regional, and city levels. More than half (n=22, 61%) of AIDSVu cities had a higher percentage for timely diagnosis than the national percentage for timely diagnosis. For receipt of care, more than half of the cities (n=24) had higher percentages than the percentage for the national average (n=661,816, 75.7%). Compared to the national percentage for viral suppression (n=565,195, 64.7%), 10 cities had higher percentages. City-to-city comparisons were made with the understanding that cities were broadly defined at the city, single county, multicounty, or MSA level.

**Table 1:** HIV care continuum indicator numerators and percentages by AIDSVu city with categorization by progress toward 2020 National HIV/AIDS Strategy goals, 36 US cities, as of 2018.

			Timely diagnosis^a^		Linkage to care^a^		Receipt of care^b^		Viral suppression^b^
NHAS^c^ goals, %			90		85		85		80
National^d^, n (%)			153,331 (78.6)		N/A^e^		661,816 (75.7)		565,195 (64.7)
**Region of participating cities, n (%)**									
	Midwest	3894 (81.1)		3715 (77.4)		14,659 (72.8)		11,412 (56.7)	
	Northeast^f^	12,144 (79.7)		1478 (75.7)		11,5136 (69.8)		98,377 (59.6)	
	South	44,040 (80.1)		33,623 (69.0)		166,653 (76.2)		132,020 (59.0)	
	West^g^	6156 (79.0)		4706 (74.0)		2714 (70.2)		23,611 (61.2)	
**City, n (%)**									
	Atlanta^h^	6206 (80.5)^i^		5321 (69.0)^i^		24,981 (75.6)^i^		20,576 (59.5)^j^	
	Austin^h^	1228 (82.0)^i^		946 (63.2)^j^		5036 (85.4)^k^		4330 (70.9)^i^	
	Baltimore^l^	2040 (77.9)^i^		1986 (75.9)^i^		13,358 (80.5)^i^		11,326 (63.1)^j^	
	Baton Rouge	788 (80.7)^i^		711 (72.8)^i^		3091 (85.7)^k^		2624 (69.7)^i^	
	Birmingham^l^	655 (78.7)^i^		576 (69.2)^i^		3299 (85.7)^k^		2757 (66.0)^i^	
	Bridgeport^h^	269 (73.3)^i^		282 (76.8)^i^		1897 (79.2)^i^		1593 (63.5)^j^	
	Charleston	344 (77.3)^i^		329 (73.9)^i^		1318 (70.3)^i^		1138 (57.0)^j^	
	Charlotte^m^	1146 (83.5)^i^		842 (61.4)^j^		4734 (78.0)^i^		4078 (61.8)^j^	
	Chicago	3395 (81.2)^i^		3227 (77.1)^i^		12,297 (75.5)^i^		9339 (54.3)^j^	
	Columbia^h^	585 (75.8)^i^		598 (77.5)^i^		1655 (66.9)^j^		1384 (51.5)^j^	
	Dallas^h^	3355 (79.6)^i^		2866 (68.0)^i^		13,675 (80.6)^i^		10,703 (59.5)^j^	
	Denver^l^	1119 (78.2)^i^		—^n^		6154 (74.0)^i^		5463 (55.4)^j^	
	Fort Lauderdale^m^	2785 (81.5)^i^		2544 (74.5)^i^		15,279 (82.6)^i^		13,285 (68.5)^i^	
	Fort Worth	1086 (77.7)^i^		893 (63.9)^j^		4295 (80.3)^i^		3587 (62.4)^j^	
	Hampton Roads^h^	1176 (80.4)^i^		932 (63.7)^j^		4607 (74.1)^i^		4213 (61.8)^j^	
	Hartford^h^	332 (73.8)^i^		344 (76.4)^i^		2385 (83.0)^i^		2093 (69.6)^i^	
	Houston	5038 (80.0)^i^		4028 (64.0)^j^		18,750 (77.2)^i^		15,471 (59.9)^j^	
	Jacksonville^h^	1266 (78.8)^i^		1020 (63.5)^j^		5611 (82.7)^i^		4412 (62.7)^j^	
	Las Vegas^m^	1662 (78.1)^i^		1715 (80.6)^i^		5965 (72.1)^i^		5149 (57.2)^j^	
	Miami^m^	5037 (82.7)^i^		4144 (68.0)^i^		18,128 (78.3)^i^		15,418 (60.1)^j^	
	Milwaukee^m^	499 (81.0)^i^		488 (79.2)^i^		2362 (85.9)^k^		2073 (70.8)^i^	
	Newark	697 (80.3)^i^		598 (68.9)^i^		2566 (56.5)^j^		1926 (62.3)^j^	
	New Haven^m^	319 (76.0)^i^		340 (81.0)^i^		2634 (87.5)^k^		1847 (62.3)^j^	
	New Orleans^l^	1256 (79.9)^i^		1103 (70.2)^i^		5130 (81.0)^i^		4421 (65.4)^i^	
	New York City	8391 (79.9)^i^		7972 (75.9)^i^		70,945 (75.8)^i^		61,702 (61.3)^j^	
	Orlando^l^	2397 (77.4)^i^		1936 (62.6)^j^		9763 (81.2)^i^		8541 (68.1)^i^	
	Philadelphia	2039 (81.8)^i^		1970 (79.0)^i^		11,711 (69.6)^i^		9832 (54.8)^j^	
	Phoenix^m^	2127 (80.9)^i^		1608 (61.2)^j^		8051 (76.8)^i^		6552 (57.4)^j^	
	Providence	97 (68.8)^i^		—^n^		347 (67.1)^j^		310 (58.1)^j^	
	Raleigh^m^	564 (79.8)^i^		461 (65.2)^j^		2685 (75.1)^i^		2285 (60.2)^j^	
	Richmond^h^	808 (81.3)^i^		658 (66.2)^j^		3496 (76.5)^i^		3066 (62.3)^j^	
	San Antonio^h^	1549 (82.3)^i^		1041 (55.3)^j^		5078 (79.8)^i^		4287 (63.9)^i^	
	Seattle^h^	1248 (77.9)^i^		1383 (86.3)^k^		6934 (85.6)^k^		6447 (77.6)^i^	
	Tampa^h^	2080 (75.9)^i^		1724 (62.9)^j^		10,442 (84.3)^i^		9120 (71.5)^i^	
	Washington DC	1527 (84.6)^i^		1296 (71.8)^i^		9293 (70.9)^i^		7748 (56.1)^j^	
	West Palm Beach^m^	1124 (75.6)^i^		950 (63.9)^j^		5600 (77.9)^i^		4865 (62.0)^j^	

^a^Timely diagnosis and linkage to care “n (%)” represents counts and percentages for 2014 through 2018.

^b^Receipt of care and viral suppression “n (%)” represents counts and percentages for 2018.

^c^NHAS: National HIV/AIDS strategy.

^d^This section represents national estimates—not all 36 cities combined.

^e^N/A: not applicable.

^f^The regional percentage for linkage to care does not include data from Providence.

^g^The regional percentage for linkage to care does not include data from Denver.

^h^Each of these cities represents a metropolitan statistical area.

^i^Within 25% of meeting NHAS goal.

^j^>25% of meeting NHAS goal.

^k^Met or surpassed NHAS goal.

^l^Each of these cities represents multiple counties.

^m^Each of these cities represents a single county.

^n^—: not available.

### Cities’ Progress Toward the HIV Care Continuum Goals

Overall, cities were closest to meeting the 2020 goals for timely diagnosis and receipt of care and struggled the most with meeting the goal for viral suppression (Table 1). The 2020 NHAS goal specified that at least 85% of people with newly diagnosed HIV should be linked to care. At the end of 2018, most of the 34 cities that provided data for linkage to care (n=20, 59%) were within 25% of the goal; 1 city (Seattle) surpassed the goal. All but 3 of the 36 cities were at least within 25% from meeting the receipt of care goal. For viral suppression, most cities (n=25, 69%) were not within 25% of the goal, and no cities met or surpassed the goal. Table S1 in Multimedia Appendix 1 contains a color-coded version of Table 1, with red, yellow, and green colors indicating categories of progress toward each of the HIV care continuum indicators’ NHAS goals.

### Cities’ Progress Toward the HIV Care Continuum Goals by Priority Populations

Table 2 displays the percentage of AIDSVu cities that were within 25% of meeting, had met, or had surpassed the NHAS goal for each HIV care continuum indicator within sex, race or ethnicity, and age groups. Overall, receipt of care was the indicator that had the highest percentage of cities that were at least within 25% of meeting the goal for select demographic groups, 97% (n=35) of cities met, surpassed, or were close to meeting the goal for females, Black people, and 13 to 24-year-olds. Viral suppression was the indicator for which most group-specific city proportions were not close to meeting the NHAS goal. Within the races and ethnicities group, the White population had the highest percentage of cities that were close to meeting, had met, or surpassed the goal for the viral suppression indicator. Only 1 demographic group—13 to 24-year-olds—had all 36 cities at least within 25% of meeting the goal for timely diagnosis. Compared to other race and ethnicity groups, the Black population had the highest percentage of cities that were close to meeting, had met, or surpassed the timely diagnosis and receipt of care goals (n=35, 97% for both). The greatest variation in percentages within a demographic category across all indicators was found for age groups. Compared to the younger age groups, older age groups had substantially lower percentages of cities whose residents had timely knowledge of their HIV+ status; however, the opposite trend was seen for the linkage to care and viral suppression indicators across age groups. Across the race and ethnicity categories, the greatest variation in percentages of cities close to meeting or having met or surpassed the NHAS goals was seen for viral suppression.

**Table 2:** Percentage of AIDSVu cities that were at least within 25% of achieving each National HIV/AIDS Strategy HIV care continuum indicator goal by sex, race or ethnicity, and age, as of 2018.

		Timely diagnosis		Linkage to care^a^		Receipt of care		Viral suppression
Overall, n (%)		36 (100)		21 (62)		33 (92)		11 (31)
**Sex, n (%)**								
	Male	35 (97)	20 (59)		32 (89)		13 (36)	
	Female	33 (92)	23 (68)		35 (97)		13 (36)	
**Race or ethnicity, n (%)**								
	Black	35 (97)	16 (47)		35 (97)		7 (19)	
	Hispanic	33 (92)	24 (71)		27 (75)		11 (31)	
	White	34 (94)	27 (79)		31 (86)		23 (64)	
**Age groups (years), n (%)**								
	13-24	36 (100)	15 (44)		35 (97)		11 (31)	
	25-44	35 (97)	22 (65)		34 (94)		5 (14)	
	45-59	7 (19)	31 (91)		32 (89)		20 (56)	
	60+	6 (17)	30 (88)		34 (94)		21 (58)	

^a^Only 34 AIDSVu cities are represented in the percentages. Denver and Providence did not provide data for linkage to care.

## Discussion

### Principal Findings

The HIV care continuum can be a useful public health tool to characterize the state of the HIV epidemic in defined areas. Much of the progress along the continuum is framed in the context of national goals, but all public health is local, and national goals will not be achieved unless they are consistently met in the cities with the largest HIV epidemics. Cities have a strong understanding of their community’s perceptions of HIV and the needs of those affected by and infected with HIV, which can be used to tailor and focus efforts. For example, in 2016 New York City clinical providers and community-based organizations created the PlaySure Network [8]. This network is dedicated to making HIV-related services such as testing, treatment, and pre-exposure prophylaxis available to all New Yorkers [8].

Here, we expand the geographic level of analysis of the HIV care continuum beyond the county-level data disseminated by the CDC [9]. A total of 27 of the 36 cities included here are located within Ending the HIV Epidemic jurisdictions [10]. The 36 cities in this analysis illustrate progress in meeting national goals overall and for particular race or ethnicity, sex, and age groups [11]. Understanding this level of patterns of inequities in access to and maintained engagement in HIV care can inform local public health action needed to improve the HIV care continuum.

Many cities publish their own surveillance reports, but different cities may use different methods to calculate key indicators. To date, a consistently derived set of indicators that surpasses the depth of the county level for at least some cities has not been available. Similarly, examining NHAS goals at the local level has a great impact. All cities were within 25% of meeting the timely diagnosis goal, but varied in how close they were to achieving the linkage to care and receipt of care goals, with 38% (n=13) of cities >25% from meeting the linkage to care goal and only 1 city that met or surpassed the linkage to care goal. Viral suppression posed the greatest challenge for cities, with only 31% (n=11) of cities falling within 25% of meeting the NHAS goal and none meeting or surpassing the goal. Among cities, there were substantial differences in meeting linkage to care and viral suppression goals among racial or ethnic groups. Finally, a higher percentage of cities were close to or met or surpassed the NHAS goals for younger populations (13 to 24 and 25 to 44-year-olds) compared to older ones (45 to 59-year-olds and those aged 60 or older) for timely diagnosis; in contrast, compared to all other age groups, the highest percentage of cities (58%, n=21) that were close to or met or surpassed the goal for viral suppression was for the oldest population (60 years of age or older). The former may reflect older people’s perception of a lower risk of HIV acquisition, while the latter may signal that younger people are less likely to stay engaged in care [12]. Additionally, younger people are unlikely to be diagnosed 8 years or more after their infection (the estimated duration of infection associated with late diagnosis) because their infection most likely happened between ages 15 and 25 years.

Among the 36 cities, there were none that achieved or surpassed the timely diagnosis NHAS goal for 2020. Thus, there is room for improvement in diagnosing people with HIV in a timely manner for cities overall. The CDC recommends that everyone aged 13 to 64 years be screened for HIV once in their lifetime, with those with certain risk factors tested annually [13]. Routine testing for those at high risk of acquiring HIV can make substantial inroads to increasing the number of timely diagnoses, but it will not allow cities to reach 100% of all those at risk of acquiring HIV. Anyone can contract HIV, so even for cities that are close to meeting the timely diagnosis goal, the last few who are not diagnosed in a timely manner are the hardest to reach. The ability of providers and patients to assess risks for HIV may not be optimal. Some studies suggest that older people are more likely to be diagnosed with advanced HIV, and women—as well as older people—have a higher likelihood of being diagnosed with HIV in a hospital [12,14]. This finding may reflect low perceived risk among these demographic groups, leading to less testing as a part of routine care. Additionally, fear of stigma may also play a role in individuals not requesting or being offered testing at younger ages or outside of hospital settings. Offering HIV tests as part of routine primary care may therefore help increase the number of timely diagnoses overall. Young people, who are often healthy, may not feel compelled to regularly engage with the health care system, so cities should focus on installing and making low-cost HIV testing services available at more accessible locations such as retail pharmacies and at home (eg, self-test kits) [15]. CDC recently announced a multiyear program to distribute HIV self-test kits directly to people who request them; this program will include direct marketing to populations with increased risks for HIV [16]. Cities will be able to incorporate referrals to the program into local prevention programming. The idea of distributing self-test kits by mail may be more acceptable after the national experience with government distribution of COVID-19 self-test kits [17].

Local, structural, and social factors can play a significant role in the ability of people with HIV to be linked to care. When there is no formal system to foster tight-knit communication between public health facilities, HIV testing sites, and HIV providers, it can be difficult to effectively link people newly diagnosed with HIV to care. Shortages of local providers who can treat people with HIV may lead to long wait times for appointments, potentially interfering with initiation of care [18-20]. It is also important to consider the local communities’ general attitudes toward HIV as they may discourage people from showing up at facilities that others know may be associated with treatment for HIV [19]. Implementation of the following has been found to promote successful linkage to care: patient navigators, mental health services, care coordination, staff training, and reduced time to provider appointments [18].

Our data highlight the importance of stratifying the HIV care continuum by demographic populations. Priority populations may differ across cities and existing prevention and care interventions may not be designed for the priority population of interest, thus requiring adaptations of the intervention. One study that constructed a dynamic HIV transmission model incorporated information regarding HIV micro epidemics within 6 differing US cities and evidence-based interventions to determine what cost-effective combination of approaches resulted in the most health benefit [21]. The researchers found that approaches needed to be tailored to each of the cities because they each had varying levels of high-risk, vulnerable populations for which some interventions worked better than others [21]. Using consistent methods of deriving HIV care continuum indicators can enable cities to discover commonalities, such as similar high-risk populations, that may promote discussions of best practices more easily.

### Limitations of the General Field

In this report, we presented the proportion of timely diagnoses among people diagnosed with HIV in 36 cities from 2014 to 2018. However, late diagnosis (the inverse of timely diagnosis) has been found to be flawed and thus, late diagnosis and timely diagnosis indicators should be used with caution because they are affected by both HIV incidence and testing [22-24]. We recommend CDC dropping these indicators and developing a new one to monitor HIV diagnosis, for example, the probability of diagnosis within 1 year of infection [25]. The timely and late diagnosis definitions are also imperfect in that they may not accurately account for those who rapidly progress to AIDS despite having tested negative for HIV within the prior year or 2.

Intermittent access to care can be difficult to isolate from the receipt of care definition because the receipt of care definition relies on testing alone. Using the CDC definition for receipt of care means that people who seek care but do not receive either test for whatever reason will be excluded.

### Limitations of the Study

Our study has several limitations. It only included data from people who were diagnosed. Thus, we do not provide insight into what populations of people are living with undiagnosed HIV. The lack of data on undiagnosed HIV means that our percentages for the 4 indicators underrepresent the true estimates. More work needs to be done to fully capture the entire population of people with HIV. Furthermore, to measure receipt of care and viral suppression, we used HIV case reporting data to estimate the denominator, that is, the number of people with HIV at the end of 2018. It has been shown that such a method can overestimate the number of people with HIV in a jurisdiction [26]. Therefore, we may have overestimated our denominator and underestimated receipt of care and viral suppression. However, because the same method was used across all 36 cities, the rankings of receipt of care and viral suppression by city and region were likely to be valid. It is recommended that HIV laboratory reporting data be used to estimate the number of people with HIV to counter the flaws with HIV case reporting data [26]. However, while this approach may lead to more accurate estimates in jurisdictions that have laws requiring full reporting of CD4 lymphocyte or viral load data, only 47 states and the District of Columbia had such laws as of December 2019 [6]. Among the 36 cities in our analysis, Newark and Philadelphia were in states that did not have such laws [6].

Cities themselves decided whether they provided input for their city alone, the county that encompassed their city, multiple counties including the one with their city, or an MSA that included their city. Cities’ data reporting was not complete, including exclusions of cases missing a zip code and exclusions due to low case count at the zip code level and for specific cities (eg, linkage to care for Denver and Providence). Additionally, not all cities had the same capacity to deduplicate their data, contributing further to the potential inflation of denominators. Also, these data do not include categories for 2 groups of people who are known to be at increased risk for HIV acquisition—men who have sex with men and people who inject drugs. Furthermore, we did not have information on nativity.

These comparisons were made with the idea that our unit of analysis, a city, was broadly defined with cities themselves deciding how their jurisdiction was defined—whether that be at the city, single county, multicounty, or MSA level. Finally, we do not include data for all major cities across the United States, such as those in California which have their own reporting mechanisms. Therefore, we are not capturing the full spectrum of progress toward the HIV Care continuum goals in all major cities or the 4 regions across the United States.

### Public Health Implications

National strategy goals cannot be achieved without first meeting these goals at the city level. Cities represent a fundamental unit for change, with cities knowing how to best reach and support the communities that dwell within their bounds. Cities have often been at the forefront of enacting fundamental policies—such as certain cities’ that make it their practice to provide pre-exposure prophylaxis at minimal cost—to prevent the transmission of HIV and improve the lives of those living with HIV. Similar to the SARS-CoV-2 vaccination for which about 67% of the US population had received at least 1 dose of the vaccine as of July 2021, with the remaining population being more difficult to vaccinate, it will also be difficult to reach the remaining people in the US population who have not received their diagnosis of HIV [27]. Advocating for primary care practices to offer HIV tests to general patient panels, rather than only high-risk patients, can facilitate timely diagnosis for all people at risk of acquiring HIV. Our data suggest that linkages to care present challenges to some cities. Cities can encourage their public health departments that may offer HIV testing to create strong ties and relationships with those that provide HIV treatment services so that there is minimal delay and a smooth transfer of patients to HIV providers. Most cities in our study seem to have some success in ensuring that people receive HIV care once they surpass the barrier of being linked to it in the first place. Our data suggest opportunities for improvement at the far end of the HIV care continuum, namely, viral suppression, which is the culmination of dedicated commitment and adequate and acceptable access to care. Those at highest risk for acquiring HIV may sometimes be some of the most vulnerable people in our cities who need the most resources to ensure that their local environment recognizes their needs and addresses them in a timely, steady, and supportive manner. With the nation’s renewed efforts to end the HIV epidemic, cities—especially high-prevalence ones—are receiving even more dedicated monies to combat this issue, making achieving national HIV care continuum goals more possible than ever before.

## Appendix

Multimedia Appendix 1 AIDSVu cities’ HIV care continuum indicators and progress toward 2020 National HIV/AIDS Strategy goals, 36 US cities, as of 2018.

## Abbreviations

CDC: Centers for Disease Control and Prevention
MSA: metropolitan statistical area
NHAS: National HIV/AIDS Strategy

## Data Availability

The data sets analyzed during this study are available from the state or local health departments on reasonable request.
