# Supplementary material for: AIDSVu Cities’ Progress Toward HIV Care Continuum Goals: Cross-Sectional Study
Source: JMIR Public Health Surveill. 2024 Feb 26;10:e49381. doi: 10.2196/49381 (PMC10928523; doi:10.2196/49381)
Supplement: Multimedia Appendix 1 [file publichealth_v10i1e49381_app1.pdf]

Multimedia Appendix 1

Table S1. AIDSVu cities' HIV care continuum indicators and progress toward 2020 National HIV/AIDS Strategy goals, 36 US cities, as of 2018.

	Timely diagnosis[a]	Linkage to care[a]	Receipt of care[b]	Viral suppression[b]
NHAS[c] goals, %	90	85	85	80
National[d], n (%)	153,331 (78.6)	N/A[e]	661,816 (75.7)	565,195 (64.7)
**Region of participating cities, n (%)**				
Midwest	3894 (81.1)	3715 (77.4)	14,659 (72.8)	11,412 (56.7)
Northeast[f]	12,144 (79.7)	1478 (75.7)	11,5136 (69.8)	98,377 (59.6)
South	44,040 (80.1)	33,623 (69.0)	166,653 (76.2)	132,020 (59.0)
West[g]	6156 (79.0)	4706 (74.0)	2714 (70.2)	23,611 (61.2)
**City, n (%)**				
Atlanta[h]	6206 (80.5)	5321 (69.0)	24,981 (75.6)	20,576 (59.5)
Austin[h]	1228 (82.0)	946 (63.2)	5036 (85.4)	4330 (70.9)
Baltimore[i]	2040 (77.9)	1986 (75.9)	13,358 (80.5)	11,326 (63.1)
Baton Rouge	788 (80.7)	711 (72.8)	3091 (85.7)	2624 (69.7)
Birmingham[i]	655 (78.7)	576 (69.2)	3299 (85.7)	2757 (66.0)
Bridgeport[h]	269 (73.3)	282 (76.8)	1897 (79.2)	1593 (63.5)
Charleston	344 (77.3)	329 (73.9)	1318 (70.3)	1138 (57.0)
Charlotte[j]	1146 (83.5)	842 (61.4)	4734 (78.0)	4078 (61.8)
Chicago	3395 (81.2)	3227 (77.1)	12,297 (75.5)	9339 (54.3)
Columbia[h]	585 (75.8)	598 (77.5)	1655 (66.9)	1384 (51.5)
Dallas[h]	3355 (79.6)	2866 (68.0)	13,675 (80.6)	10,703 (59.5)
Denver[i]	1119 (78.2)	—[k]	6154 (74.0)	5463 (55.4)
Fort Lauderdale[j]	2785 (81.5)	2544 (74.5)	15,279 (82.6)	13,285 (68.5)
Fort Worth	1086 (77.7)	893 (63.9)	4295 (80.3)	3587 (62.4)
Hampton Roads[h]	1176 (80.4)	932 (63.7)	4607 (74.1)	4213 (61.8)
Hartford[h]	332 (73.8)	344 (76.4)	2385 (83.0)	2093 (69.6)
Houston	5038 (80.0)	4028 (64.0)	18,750 (77.2)	15,471 (59.9)
Jacksonville[h]	1266 (78.8)	1020 (63.5)	5611 (82.7)	4412 (62.7)
Las Vegas[j]	1662 (78.1)	1715 (80.6)	5965 (72.1)	5149 (57.2)
Miami[j]	5037 (82.7)	4144 (68.0)	18,128 (78.3)	15,418 (60.1)
Milwaukee[j]	499 (81.0)	488 (79.2)	2362 (85.9)	2073 (70.8)
Newark	697 (80.3)	598 (68.9)	2566 (56.5)	1926 (62.3)
New Haven[j]	319 (76.0)	340 (81.0)	2634 (87.5)	1847 (62.3)
New Orleans[i]	1256 (79.9)	1103 (70.2)	5130 (81.0)	4421 (65.4)

New York City	8391 (79.9)	7972 (75.9)	70,945 (75.8)	61,702 (61.3)
Orlando[i]	2397 (77.4)	1936 (62.6)	9763 (81.2)	8541 (68.1)
Philadelphia	2039 (81.8)	1970 (79.0)	11,711 (69.6)	9832 (54.8)
Phoenix[j]	2127 (80.9)	1608 (61.2)	8051 (76.8)	6552 (57.4)
Providence	97 (68.8)	—[k]	347 (67.1)	310 (58.1)
Raleigh[j]	564 (79.8)	461 (65.2)	2685 (75.1)	2285 (60.2)
Richmond[h]	808 (81.3)	658 (66.2)	3496 (76.5)	3066 (62.3)
San Antonio[h]	1549 (82.3)	1041 (55.3)	5078 (79.8)	4287 (63.9)
Seattle[h]	1248 (77.9)	1383 (86.3)	6934 (85.6)	6447 (77.6)
Tampa[h]	2080 (75.9)	1724 (62.9)	10,442 (84.3)	9120 (71.5)
Washington D.C.	1527 (84.6)	1296 (71.8)	9293 (70.9)	7748 (56.1)
West Palm Beach[j]	1124 (75.6)	950 (63.9)	5600 (77.9)	4865 (62.0)

Color coding: green—met or surpassed NHAS goal; yellow—within 25% of meeting NHAS goal; red—> 25% from meeting NHAS goal

[a]Timely diagnosis and linkage to care "n (%)" represents counts and percentages for 2014 through 2018.

[b]Receipt of care and viral suppression "n (%)" represents counts and percentages for 2018.

[c]NHAS: National HIV/AIDS strategy.

[d]This section represents national estimates—not all 36 cities combined.

[e]N/A: not applicable.

[f]The regional percentage for linkage to care does not include data from Providence.

[g]The regional percentage for linkage to care does not include data from Denver.

[h]Each of these cities represents a metropolitan statistical area.

[i]Each of these cities represents multiple counties.

[j]Each of these cities represents a single county.

[k]—: not available.
